# Thrombosis as the First Manifestation of Granulomatosis with Polyangiitis Disease in an Adolescent

**DOI:** 10.1155/2021/5520258

**Published:** 2021-03-03

**Authors:** Mohsen Jari

**Affiliations:** Department of Pediatric Rheumatology, Imam Hossein Children's Hospital, Isfahan University of Medical Sciences, Isfahan, Iran

## Abstract

**Background:**

Granulomatosis with polyangiitis disease (GPA) is a rare vasculitis characterized by granulomatous inflammation of respiratory tracts, glomerulonephritis, and vasculitis of other organs. *Case Presentation*. A 13-year-old girl was referred due to swelling and pain on her left arm. The Doppler and compression ultrasonography showed noncompressible left brachial and axillary vein thrombosis. Sinus computed tomography (CT) demonstrated pansinusitis, and spiral chest CT showed alveolar hemorrhage. Laboratory tests showed hematuria, proteinuria, and highly positive antineutrophil cytoplasmic antibody (cANCA). Laboratory tests of coagulopathy were normal. The patient was recognized as a case of GPA.

**Conclusion:**

Although GPA is not frequently associated with thrombosis especially in children, this is the first report that shows thrombosis may be the first manifestation of GPA in an adolescent.

## 1. Introduction

Thrombosis can occur in the artery or vein. Venous thrombosis is less common in children than in adults, but leads to more morbidity and mortality that emphasize timely diagnosis and therapy [1, 2]. Risk factors for thrombosis include central venous catheter (CVC), immobility, heart disease, coagulation disorders, congenital heart disease, trauma (especially fractures), cancer, surgery, infections, dehydration, shock, obesity, nephrotic syndrome, antiphospholipid antibody syndrome, and vasculitis [1–3]. Granulomatosis with polyangiitis disease (GPA), formerly Wegener's granulomatosis, is a chronic vasculitis. Thrombosis is a rare manifestation of this disease especially in children and adolescents [2, 3].

## 2. Case Presentation

A 13-year-old girl was admitted to the pediatric rheumatology ward of Imam Hossein Children's Hospital, Isfahan University of Medical Sciences, due to swelling and pain on her left arm 3 days prior to admission. Since 3 months, she had fatigue, bloody nasal discharge, and cough.

On physical examination, she was alert, and her blood pressure and temperature were within normal limit. Her body weight was 43 kg. There were no abnormalities on heart and lung examination. There was no lymphadenopathy or hepatosplenomegaly. There was swelling on her left arm without any sign of inflammation. The size of her left vs. right arm and left vs. right forearm was 28 cm vs. 23 cm and 18 cm vs. 17 cm, respectively.

The radial and brachial pulses on the left were reduced. The laboratory examinations showed anemia (hemoglobin, 8.3 g/dL); leukocytosis (white blood count, 19500/mm^3^ (lymphocytes 24%)); thrombocytosis (platelets = 530.000/mm^3^); elevation of estimated sedimentation rate (ESR = 105 mm/hr) and C-reactive protein (CRP = 97 mg/L); prothrombin time (PT), 13.0 seconds; INR, 1.1; activated partial thromboplastin time (aPTT), 32.3 seconds; and d-dimer 5.8 mg/L (normal <0.3 mg/L). The serum levels of protein C, protein S, antithrombin III, and factor V Leiden were normal. Renal function and electrolytes were within normal limits. Peripheral blood smear was normal.

Urinalysis showed proteinuria 3+ and hematuria 4+, and 24-hour urine showed proteinuria (750 mg/24 hrs). Antinuclear antibody (ANA) was negative, and antiphospholipid antibodies, anti-DNA antibody, and serum complement levels were normal. Antineutrophil cytoplasmic antibody (ANCA) was highly positive (cANCA = 450 U/ml, Nl <25 U/ml).

The Doppler and compression ultrasonography showed noncompressible and completely occlusive thrombosis of left brachial and axillary veins (noncompressible venous segment, lack of flow augmentation with calf squeeze, and increased flow in superficial veins). Sinus computed tomography (CT) demonstrated mucosal thickening and pansinusitis, and spiral chest CT showed minimal alveolar hemorrhage.

According to the EULAR/PRINTO//PRES criteria, the patient was recognized as a case of GPA, and the treatment started by prescribing subcutaneous enoxaparin and pulse methylprednisolone 30 mg/kg/d (max 1 gr) for three days. Then, in the fourth day of treatment, intravenous infusion (IV) with cyclophosphamide 500 mg/m^2^ was given. The patient was discharged in the fifth day given oral prednisolone 2 mg/kg/d and enoxaparin. After a one-week follow-up, the Doppler and compression ultrasonography of left brachial and axillary veins was normal. Enoxaparin was continued (for three months), and IV cyclophosphamide, oral prednisolone, and azathioprine were continued based on EULAR/PRINTO//PRES criteria.

## 3. Discussion

Granulomatosis with polyangiitis disease (GPA) is not frequently associated with thrombosis. There are few reports of intracranial thrombosis of large and small vessels in adults and children. These events have been occurred due to intracranial vasculitis or extension of granulomatous lesions from the nasal cavity [1, 2]. Few recent reports of thrombosis at other organs have been described in some adults, that are very rare in children and adolescents [3–6]. Systematic studies of factors contributing to hypercoagulability in these patients were not reported.

GPA is a antineutrophil cytoplasmic antibody- (ANCA-) associated vasculitis (AAV) characterized by necrotizing inflammation of small vessels and the presence of ANCA directed to specific antigens, particularly proteinase 3 (PR3-ANCA) and myeloperoxidase (MPO-ANCA). This disease [7] primarily affects the small vessels of the skin, lungs, and kidneys. During AAV, proinflammatory cytokines are elevated in the serum of these patients.

Circulating tumor necrosis factor (TNF) is a major factor of atherothrombotic events in AAV [8, 9].

Pathogenesis of thrombosis in AAV including endothelial cell dysfunction and interaction between neutrophils (activated by TNF*α* and ANCA) and with consequent massive oxidative stress finally lead to atherothrombotic complications [10]. TNF can increase gene expression of procoagulant proteins, such as tissue factor. Anti-PR3 antibodies can increase the expression of tissue factor [11]. Recently, reports demonstrated that neutrophils are able to release extracellular nucleic acids associated with histones and granular proteins capable of entrapping bacterial agents [12, 13].

These mechanisms may explain why in patients with GPA thrombosis occurred during the initial stage of disease or when inflammation was not yet controlled.

## 4. Conclusion

Although GPA and other AAV are not frequently associated with thrombosis especially in children and adolescents, thrombosis may be the first manifestation of GPA.

## Data Availability

The data used to support the findings of this study are available from the author upon request.
